# Artificial intelligence in a Brazilian lung cancer screening program: accuracy and predictive values

**DOI:** 10.31744/einstein_journal/2025AO1283

**Published:** 2025-11-07

**Authors:** Ricardo Sales dos Santos, Gustavo Borges da Silva Teles, Rodrigo Caruso Chate, Gilberto Szarf, Cesar Augusto de Araújo, Juliana Pereira Franceschini, Mário Claudio Ghefter, Ivan Drokin, Marcos Duarte Guimarães, Bruno Hochhegger

**Affiliations:** 1 Instituto ProPulmão Salvador BA Brazil Instituto ProPulmão, Salvador, BA, Brazil.; 2 Hospital Israelita Albert Einstein São Paulo SP Brazil Hospital Israelita Albert Einstein, São Paulo, SP, Brazil.; 3 Universidade SENAI CIMATEC Salvador BA Brazil Universidade SENAI CIMATEC, Salvador, BA, Brazil.; 4 Universidade Federal da Bahia Salvador BA Brazil Universidade Federal da Bahia, Salvador, BA, Brazil.; 5 Fundação ProAR Salvador BA Brazil Fundação ProAR, Salvador, BA, Brazil.; 6 Hospital do Servidor Público Estadual "Francisco Morato de Oliveira" Thoracic Surgery Department São Paulo SP Brazil Thoracic Surgery Department, Hospital do Servidor Público Estadual "Francisco Morato de Oliveira", São Paulo, SP, Brazil.; 7 Territory of the Skolkovo Innovation Centre Intellogic LCC Moscow Russia Intellogic LCC, Territory of the Skolkovo Innovation Centre, Moscow, Russia.; 8 Universidade Federal do Vale do São Francisco Petrolina PE Brazil Universidade Federal do Vale do São Francisco, Petrolina, PE, Brazil.; 9 University of Florida Gainesville USA University of Florida, Gainesville, USA.

**Keywords:** Lung neoplasms, Mass screening, Tomography, x-ray computed, Radiation dosage, Artificial intelligence

## Abstract

**Objective:**

We aimed to investigate the performance of an artificial intelligence (AI)-powered radiology platform for detecting pulmonary nodules on low-dose computed tomography of the chest compared with expert radiologists. The ancillary aims were to assess the Lung-RADS category agreement between the software and radiologists and to evaluate the percentage of missed lung nodules >6mm or those with inappropriate segmentation in the analysis performed by the AI software.

**Methods:**

This was a cross-sectional study. We evaluated low-dose computed tomography scans of 790 patients enrolled in a lung cancer screening program. All computed tomography scans were reviewed by an experienced team of thoracic radiologists (expert group). An AI algorithm analyzed the same set of scans independently, anonymously, and blinded to the computed tomography results (AI Group). The Lung-RADS classification system was used for both groups, and the reported findings were compared, considering expert analysis as the gold standard. Therefore, the accuracy results, negative and positive predictive values of AI, were measured.

**Results:**

The AI Group software showed high sensitivity (92.5%) and negative predictive value (97.8%) but low specificity (78.5%) and positive predictive value (50%). A significant number of subsolid nodules were missed in the AI group; however, none of them were >8mm (Lung-RADS 4).

**Conclusion:**

The AI software demonstrated a high negative and relatively low positive predictive values. The device appears to be an important adjunct, allowing the navigation team to prioritize examinations for clinically significant nodules.

## In Brief

We evaluated an artificial-intelligence-powered platform for detecting lung nodules on low-dose computed tomography scans of 779 high-risk individuals using a Brazilian screening program. The artificial intelligence demonstrated high sensitivity and negative predictive value, suggesting its potential as a tool for prioritizing clinically significant findings.

## Highlights

■ The artificial intelligence tool demonstrated high sensitivity (92.5%) and a negative predictive value of 97.8%. This effectively excluded examinations that were not clinically significant.
■ However, its low specificity (78.5%) and positive predictive value (50%) emphasize the importance of radiologist's supervision.
■ - artificial intelligence missed 4.7% of nodules measuring >6mm, most of which were subsolid (62%).
■ The tool has the potential to improve workflows in lung cancer screening programs.

## Graphical abstract

**Figure:**
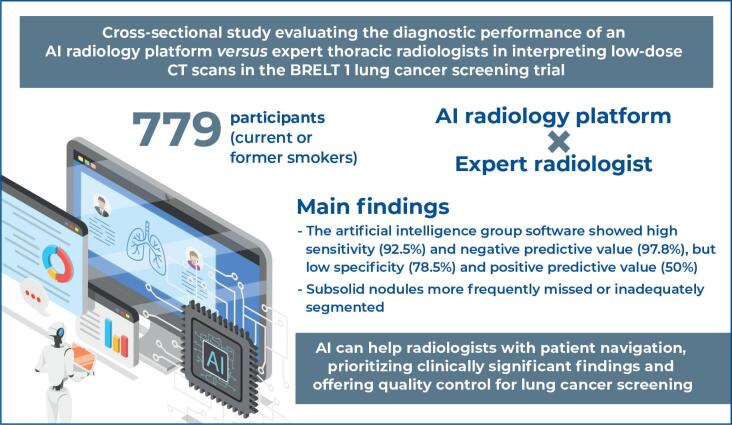

## INTRODUCTION

Lung cancer is one of the most common types of cancer and remains the leading cause of cancer-related deaths in Brazil.^(1)^ The prevalence of lung cancer has been decreasing among men in Brazil, mainly because of the significant decline in the percentage of adult smokers over the past 20 years. In contrast, it has increased in the female population, predominantly among never-smokers.^(2,3)^

Despite significant advances in the diagnosis and treatment of lung cancer, the disease is still associated with poor clinical outcomes, and survival strongly depends on the stage of diagnosis.^(4)^ Early detection using low-dose computed tomography (LDCT) screening can change this scenario and reduce lung cancer mortality. Since the results of the National Lung Screening Trial (NLST)^(5)^ showed a significant decrease in the lung cancer-specific mortality rate, recommendations for screening have been evolving around the world.^(6)^

Although LDCT has improved the detection rate, it also increases the workload of radiologists.^(7)^ Several studies have used artificial intelligence (AI) tools to develop algorithms capable of identifying imaging features from LDCT scans that may be specific to lung cancer. Applying AI technology to the preliminary screening of LDCT images and marking suspicious lesions can reduce workload and improve diagnostic accuracy, which could improve lung cancer screening.^(8)^

Artificial intelligence in diagnostic radiology is rapidly developing. Recent studies have shown that AI can improve the detection and characterization of pulmonary nodules and reduce the reading times.^(8–11)^

## OBJECTIVE

We investigated the performance of an artificial-intelligence-powered radiology platform for detecting pulmonary nodules on low-dose computed tomography scans. It also assessed Lung-RADS category agreement between the software and radiologists and evaluated the percentage of missed nodules.

## METHODS

This was a cross-sectional study. We evaluated all LDCT studies performed at our institution as part of the Brazilian Lung Cancer Screening Trial (BRELT 1)^(12)^ between January 2013 and December 2014. The inclusion criteria were current or former heavy smokers (≥30 pack-years, with no more than 15 years of smoking abstinence) between the ages of 55 and 74, and the absence of significant respiratory symptoms. Exclusion criteria included inability to undergo computed tomography (CT), pregnancy, prior radiation therapy to the chest, and severe comorbidities such as cardiovascular, pulmonary, hepatic, renal, or metabolic diseases. This study was approved by the local Institutional Review Board of the *Hospital Israelita Albert Einstein* CAAE: 40382720.9.0000.0071; 4.791.870.

### Image acquisition

All CT examinations were performed without intravenous contrast injection, using a 64-row multidetector CT scanner (Toshiba Aquilion 64, Toshiba Medical Systems, Tokyo, Japan) with a low-dose technique (120kV, 15mAs maximum) and the adaptive iterative dose reduction feature. Volumetric helical CT scans of the thorax were obtained. Images were reconstructed with 1mm section thickness and 1mm intervals, using a high spatial frequency (lung) and soft reconstruction algorithm, and stored in the Digital Imaging and Communications in Medicine format. These image acquisition methods were defined in the BRELT 1 study,^(12)^ which was previously published.

### AI analysis: description of the AI neural network process

The conventional pipeline for Computed Aid Diagnosis (CAD) screening typically consists of multiple stages, primarily involving the detection and classification of cancer. Initially, a nodule detector was used to identify the nodules within the scan, followed by an assessment to determine malignancy. In this study, we propose an end-to-end approach for both stages. Specifically, we developed and integrated the detector and false-positive reduction stages into a single convolutional neural network. The proposed architecture is based on a trainable version of the maximum intensity projection (MIP) block,^(13)^ which serves as an initial feature extractor, followed by a U-Net-like segmentation network. This end-to-end approach eliminates the necessity for specific data sources or annotations but requires some data preparation. We outline an evaluation process that includes comparisons with the current state-of-the-art method. A more comprehensive description of the proposed method was published by Drokin et al.^(14)^

### Overall pipeline description: model-based feature projection

A framework using semantic segmentation for lung nodule detection was proposed. Instead of relying only on axial images from CT scans, sagittal and coronal projections were also used to improve the accuracy of analyzing complex findings. This approach facilitated the training of a more robust model without expanding the training dataset.^(15)^ During inference, sagittal and coronal projections were prepared, and the model was inferred independently on each slice. Predictions were then averaged by transforming the data back to axial projections, providing a more accurate representation of the three-dimensional shape of the findings and reducing false positives.^(16–19)^

### Experiment on LUNA2016

To evaluate the framework, we used the LUNA2016 dataset. Computed tomography scans were resampled to 0.8mm spacing, and networks were trained with all three projections simultaneously. The proposed framework outperformed recently published results, particularly in detecting nodules >5mm, with low false-positive rates.

Visualization of the features fed into the segmentation network revealed that the model-based features effectively ignored normal lung tissue, preserving valuable information about nodules, while reducing false positives caused by structures such as bronchi and blood vessels. In addition, the proposed model-based feature projection blocks demonstrated better noise suppression than the MIP projections from the CT scanners.^(20–22)^

### Radiologists’ CT analysis

Two board-certified thoracic radiologists (with 14 and 2 years of experience in interpreting chest images), blinded to the results of the AI software, reviewed all anonymized chest CT images independently in a standard clinical Picture Archiving and Diagnostic System workstation. They classified the examinations according to Lung-RADS 1.1 categories^(23)^ and assessed all lung nodules >6mm (mean diameter). The final classification was based on consensus between the readers.

After the initial reading, the thoracic radiologist reviewed the analysis performed using the AI software for all examinations. The number and characteristics (solid *versus* subsolid, subpleural *versus* nonsubpleural location) of lung nodules >6mm not detected by the AI software and the number and characteristics of nodules >6mm with inadequate segmentation performed by the AI platform were recorded.

### Statistical analyses

All statistical analyses were performed using the IBM SPSS Statistics for Windows, version 22 (IBM Corp., Armonk, NY, USA). Data are reported as the mean±standard deviation or number (%) unless otherwise indicated.

To determine and compare the performance of Lung-RADS between the radiologist and the AI algorithm, the Kendall rank correlation coefficient was calculated. Images were categorized as 3 and 4 *versus* 1, 2, and 4 *versus* 1, 2, and 3. Sensitivity, specificity, positive predictive value (PPV), and negative predictive value (NPV) were calculated using Kappa coefficients with 95% confidence intervals. In addition, the sensitivity and specificity of lung cancer classification were analyzed based on the results of the area under the receiver operating characteristic (ROC) curve analyses to assess any bias in agreement.

In all analyses, statistical significance was set at p<0.05.

## RESULTS

Between January 2013 and December 2014, 790 patients were screened, of whom nine (1.13%) had lung cancer. These results were reported in BRELT 1.^(12)^

Table 1 shows diagnostic performance and agreement (negative *versus* positive LDCT). The AI group showed good diagnostic performance with moderate agreement with the radiologists’ evaluations. The expert group classified 147 LDCTs (19%) as positive, whereas AI group classified 272 CTs (35%) as positive. Only 11 LDCTs (1.4%) were classified as positive by the experts but as negative by the AI group. The AI software demonstrated high sensitivity and NPV (97.8%) but low specificity and PPV (56.1%) (Table 2). Precision (equivalent to PPV formula) was 50% (95% confidence interval [95%CI]: 43.9-56.1), accuracy 81.2% (95% CI: 78.5-83.9), and F1-score 64.9% (95% CI: 61.6-68.3).

**Table 1:** Diagnostic performance and agreement (negative *versus* positive CT)

		AI platform		Total n (%)	Kappa (95%CI)	Sensitivity (95%CI)	Specificity (95%CI)	AUC ROC (95%CI)	PPV (95%CI)	NPV (95%CI)
		Negative CT n (%)	Positive CT n (%)							
Radiologist										
	Negative CT	496 (63.7)	136 (17.5)	632 (81.1)	0.535	92.5	78.5	0.855	50	97.8
	Positive CT	11 (1.4)	136 (17.5)	147 (18.9)	(0.474; 0.596)	(87; 96.2)	(75.1; 81.6)	(0.828; 0.882)	(43.9; 56.1)	(96.2; 98.9)
	Total	507 (65.1)	272 (34.9)	779 (100)						

Negative CT, Lung RADS 1 and 2; positive CT, Lung RADS 3 and 4. CT: computed tomography; 95%CI: 95% confidence interval; PPV: positive predictive value; NPV: negative predictive vvalue; AI: artificial intelligence.

**Table 2:** Lung-RADS categories agreement

Lung-RADS		AI platform					Total n (%)	Correlation Coefficient Tau-b Kendall
		1 n (%)	2 n (%)	3 n (%)	4A n (%)	4B n (%)		
Radiologists								
	1	198 (25.4)	1 (0.1)	3 (0.4)	5 (0.6)	2 (0.3)	209 (26.8)	0.590
	2	227 (29.1)	70 (9.0)	92 (11.8)	28 (3.6)	6 (0.8)	423 (54.3)	
	3	5 (0.6)	5 (0.6)	57 (7.3)	23 (3.0)	5 (0.6)	95 (12.2)	
	4A	0 (0.0)	0 (0.0)	1 (0.1)	36 (4.6)	5 (0.6)	42 (5.4)	
	4B	1 (0.1)	0 (0.0)	0 (0.0)	1 (0.1)	6 (0.8)	8 (1.0)	
	4X	0 (0.0)	0 (0.0)	0 (0.0)	1 (0.1)	1 (0.1)	2 (0.3)	
	Total	431 (55.3)	76 (9.8)	153 (19.6)	94 (12.1)	25 (3.2)	779 (100)	

AI: artificial intelligence.

Conversely, the AI software detected 784 nodules >6mm; in contrast, the software missed 39 nodules (4.7%) between 6 and 44mm, with 62% subsolid. Of the 784 detected nodules, 86 were evaluated with inappropriate segmentation (11%), and 97% were solid (predominantly subpleural).

**Figure 1:**
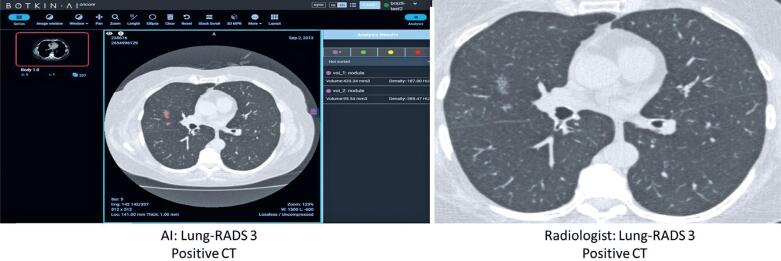
Agreement between the artificial intelligence software and radiologist in a pulmonary nodule classified as Lung-RADS 3

AI: artificial intelligence; CT: computed tomography.

**Figure 2:**
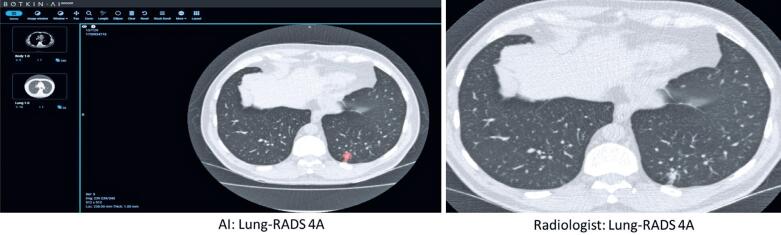
Agreement between the artificial intelligence software and radiologist in a pulmonary nodule classified as Lung-RADS 4A

**Figure 3:**
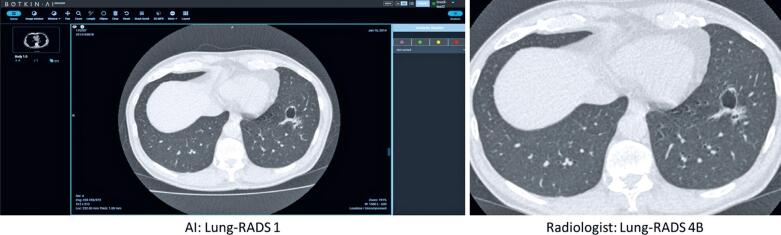
Discordant classification: Nodule scored as Lung-RADS 1 by artificial intelligence *versus* Lung-RADS 4B by radiologist

**Figure 4:**
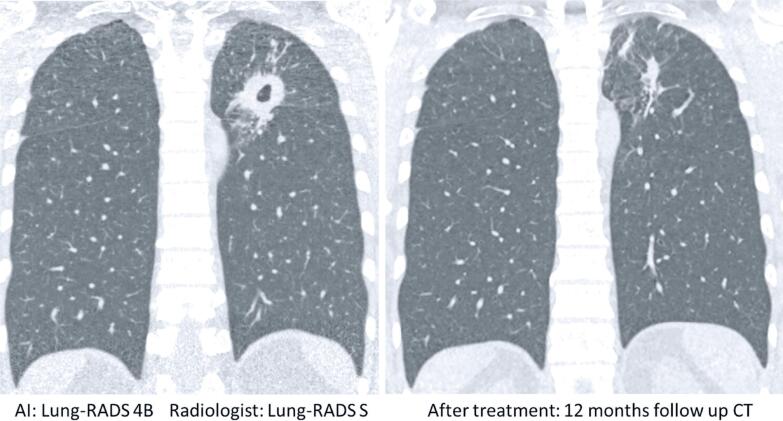
Discordant classification: Nodule identified as Lung-RADS 4B by artificial intelligence *versus* Lung-RADS S in radiologist's assessment

AI: artificial intelligence; CT: computed tomography.

Lung cancer cases are shown in table 3, comparing data from the AI software and radiologists’ evaluation. Figure 1 shows the agreement between the AI software and radiologist for a pulmonary nodule classified as Lung-RADS 3. Figure 2 also shows the agreement between the AI software and radiologist for a pulmonary nodule classified as Lung-RADS 4A, which was later confirmed by biopsy as an adenocarcinoma of the lung. By contrast, figures 3 and 4 highlight the discrepancies between the two assessments. In figure 3, the AI software classified the exam as negative, whereas the radiologist interpreted it as Lung-RADS 4B; subsequent follow-up with positron emission tomography/ CT showed no abnormalities, and the lesion remained unchanged on the 12-month follow-up CT. In figure 4, the AI software classified the findings as Lung-RADS 4B, whereas the radiologist interpreted them as Lung-RADS S. Subsequent bronchoscopy confirmed tuberculosis, and the 12-month follow-up CT demonstrated significant improvement after treatment.

**Table 3:** Lung-RADS categories in diagnosed lung cancer cases

Patient	Lung-RADS radiologist	Lung-RADS AI platform	Diagnosis	Staging
1	4A	4B	Invasive squamous cell carcinoma	pT1a pN0
2	4B	4B	Adenocarcinoma of the lung	pT1a pN0
3	4B	4A	Adenocarcinoma of the lung	pT1a pN0
4	4B	4B	Adenocarcinoma of the lung	pT1a pN0
5	4A	4A	Squamous cell carcinoma	pT1b pN0
6	4X	4B	Nonsmall cell lung cancer	ypT1a ypN0
7	4B	4B	Invasive adenocarcinoma of the lung	pT2a pN0
8	4A	4A	Adenocarcinoma of the lung	pT1a pN0
9	3	3	Carcinoid tumor	

AI: artificial intelligence.

AI: artificial intelligence.

AI: artificial intelligence.

## DISCUSSION

Models for predicting malignancy in pulmonary nodules detected using LDCT consider the most commonly described radiological variables, such as size, location, presence of spicules, and emphysema.^(24)^ The ability to discriminate the status of malignancy is generally related to the area under the curve (AUC), with the ROC curve derived from the traditional elements of accuracy measurement, sensitivity, and specificity.^(25)^

Deep-learning algorithms can diagnose certain pathologies on chest radiographs at a level comparable to that of radiologists. In a study of 14 clinically important pathologies, the algorithm was able to locate the region most indicative of the disease with equivalent performance in 10 pathologies, better in one, and worse in three. However, the radiologists classified 420 images in 240 min, whereas the algorithm completed in 1.5 min.^(26)^ These results highlight the potential of AI for enhancing the diagnostic efficiency of large-scale screening programs.

However, the ability of computers to evaluate such data is limited. Some studies using AI tools have evaluated several databases, such as the Multicentric Italian Lung Detection, Danish Lung Cancer Screening Trial, and NLST. Deep learning methodologies, dynamic Bayesian networks, and convolutional neural networks have been used specifically for lung cancer to differentiate between benign and cancerous nodules more accurately and thus improve lung cancer screening. The average AUC results were above 0.75, with sensitivity and specificity >83%, leading the doctors to conclude that the models were comparable to those of experienced radiologists, even with subgroup analysis of small nodules (<10mm).

Previous studies have shown that chest radiography is not effective in reducing lung cancer mortality when used as a screening method. However, deep convolutional neural network models have outperformed physicians, including thoracic radiologists, in CXRs analysis and the evaluation of malignant pulmonary nodules. Computer models demonstrated higher sensitivity than radiologists for detecting operable lung cancer with CXRs, suggesting that CXR coupled with AI may have potential value in lung cancer screening.^(8)^

This is the first study to use an AI tool to analyze an image database obtained from a structured lung cancer screening program in Brazil.

A three-dimensional deep convolutional neural network model achieved an AUC of 94.4% when analyzing 6716 NLST cases. The model performed comparably to six thoracic radiologists; however, it was superior, with absolute reductions of 11% in false positives and 5% in false negatives when prior CT images were not available.^(27)^

The software (AI platform) demonstrated high sensitivity and NPV, providing a low rate of missed nodules (>6mm), and is potentially useful as a triage tool to support radiologists in the quality control of a large number of images obtained for early lung cancer screening. Notably, this software did not miss any images classified as Lung-RADS 4 by radiologists, indicating its potential applicability for central navigation of participants in tracking programs in Brazil.

Considerable variability in the sensitivity of pulmonary nodule detection by CAD systems has been reported in previous studies, ^(28–31)^ ranging from 38% in a study by Wormanns et al.^(29)^ to 84% in Armato et al.^(28)^ The false-negative rates of CAD systems limit their application as a stand-alone technique, primarily because of limitations related to nodule size (small nodules <4mm), attenuation (subsolid nodules, particularly pure ground-glass nodules), and segmentation algorithms, as CAD systems more readily recognize nodules surrounded by lung parenchyma, potentially struggling with subpleural lesions adjacent to the chest wall structures. Additionally, the false-positive rates of CAD systems range 3-13 nodules per CT scan in these studies, with pulmonary vessels and scars being among the main causes of nodule misinterpretation.

In a study evaluating the performance of a CAD system compared with that of a radiologist in detecting pulmonary nodules on 150 LDCT scans for lung cancer screening, the radiologist detected 518 (82%) of 628 true nodules, whereas the CAD system detected 456 (73%) of 628 true nodules.^(32)^ Moreover, the CAD system identified additional 478 lesions that were classified as false-positive nodules upon radiologist review, resulting in a rate of 3.19 (478/150) per patient. However, the radiologist failed to identify 110 true nodules that were detected exclusively by the CAD system. In six patients, these were the only nodules detected on the scan, altering the imaging follow-up protocol. Therefore, the combined evaluation of LDCT scans by both the radiologist and CAD system was necessary to identify all nodules. The results also demonstrated a complementary interaction between the CAD system and the radiologist across different lung regions. Radiologists tend to have little difficulty identifying peripheral and subpleural nodules, even when small, because of the absence of similarly sized vessels near the pleural surface. In contrast, CAD systems are more sensitive in detecting central nodules, particularly hilar nodules located among large vessels, which are more likely to be mistaken for vessels and consequently overlooked by radiologists.

The limitations of this software include its low specificity. In this study, the PPV of the software was <60%, indicating that a significant number of nodules could not be confirmed by a radiologist. This limitation initially implies an increase in the radiologist's workload. Therefore, the instrument should be useful for prioritizing potentially suspicious scans, placing those that were considered negative at the end of the queue. This method is safe because a high NPV of >97% was verified. The segmentation of subpleural nodules represents a significant challenge for AI algorithms because of the proximity of these lesions to the pleura, which makes it difficult to precisely define their contours. Previous studies have indicated that detecting these lesions can be limited in AI systems owing to reconstruction artifacts and poor differentiation from adjacent structures such as blood vessels and scar tissue.

Another key aspect warranting further investigation is the analysis of discordant cases, particularly among clinically significant categories, such as Lung-RADS 4. Although the AI model identified more positive cases than radiologists, understanding the clinical relevance of these disagreements is crucial. The high rate of false positives observed in AI analysis can significantly impact the workflow of radiologists, generating a larger number of examinations for review and potentially increasing the workload of specialists. Future studies should investigate whether AI-classified positive nodules missed by radiologists represent early-stage cancers or benign lesions, leading to unnecessary follow-up. Similarly, evaluating false-negatives, particularly subsolid nodules, is essential for refining AI performance and ensuring that it aligns with clinical needs.

External validation of the model was performed using the LUNA2016 dataset, an internationally recognized image database widely used in lung nodule detection studies. However, we acknowledge significant differences between the LUNA2016 population and our study cohort, including variations in image quality, acquisition protocol, and prevalence of pulmonary diseases such as granulomatous disease, which is more common in Brazil. These differences may have affected the generalizability of our results.

Another limitation was the difficulty in segmenting subpleural lung nodules and the inability to detect other clinically significant findings (nonlung cancer). This software was also less accurate for nonsolid nodules, which represents an area for future improvement.

## CONCLUSION

The artificial intelligence software demonstrated a high negative and relatively low positive predictive value. The device may serve as an important adjunct to help navigation teams prioritize examinations for clinically significant nodules.

## References

[1] Araujo LH, Baldotto C, Castro G, Katz A, Ferreira CG, Mathias C, Mascarenhas E, Lopes GL, Carvalho H, Tabacof J, Martínez-Mesa J, Viana LS, Cruz MS, Zukin M, Marchi P, Terra RM, Ribeiro RA, Lima VCC, Werutsky G, Barrios CH, Grupo Brasileiro de Oncologia Torácica (2018). Lung cancer in Brazil. J Bras Pneumol.

[2] Instituto Nacional de Câncer José Alencar Gomes da Silva (INCA) (2019). Estimativa 2020: incidência de câncer no Brasil.

[3] Mathias C, Prado GF, Mascarenhas E, Ugalde PA, Zimmer Gelatti AC, Carvalho ES, Faroni LD, Oliveira R, Cordeiro de Lima VC, de Castro G, Grupo Brasileiro de Oncologia Torácica (2020). Lung Cancer in Brazil [editorial]. J Thorac Oncol.

[4] de Koning HJ, van der Aalst CM, de Jong PA, Scholten ET, Nackaerts K, Heuvelmans MA (2020). Reduced Lung-Cancer Mortality with Volume CT Screening in a Randomized Trial. N Engl J Med.

[5] National Lung Screening Trial Research Team; Aberle DR, Adams AM, Berg CD, Black WC, Clapp JD, Fagerstrom RM, Gareen IF, Gatsonis C, Marcus PM, Sicks JD (2011). Reduced lung-cancer mortality with low-dose computed tomographic screening. N Engl J Med.

[6] Arenberg D (2019). Update on screening for lung cancer. Transl Lung Cancer Res.

[7] Li X, Guo F, Zhou Z, Zhang F, Wang Q, Peng Z (2019). [Performance of Deep-learning-based Artificial Intelligence on Detection of Pulmonary Nodules in Chest CT]. Zhongguo Fei Ai Za Zhi.

[8] Espinoza JL, Dong LT (2020). Artificial Intelligence Tools for Refining Lung Cancer Screening. J Clin Med.

[9] Liu K, Li Q, Ma J, Zhou Z, Sun M, Deng Y (2019). Evaluating a Fully Automated Pulmonary Nodule Detection Approach and Its Impact on Radiologist Performance. Radiol Artif Intell.

[10] Causey JL, Zhang J, Ma S, Jiang B, Qualls JA, Politte DG (2018). Highly accurate model for prediction of lung nodule malignancy with CT scans. Sci Rep.

[11] Joy Mathew C, David AM, Joy Mathew CM (2020). Artificial Intelligence and its future potential in lung cancer screening. EXCLI J.

[12] dos Santos RS, Franceschini JP, Chate RC, Ghefter MC, Kay F, Trajano AL (2016). Do Current Lung Cancer Screening Guidelines Apply for Populations With High Prevalence of Granulomatous Disease? Results From the First Brazilian Lung Cancer Screening Trial (BRELT1). Ann Thorac Surg.

[13] Wallis JW, Miller TR, Lerner CA, Kleerup EC (1989). Three-dimensional display in nuclear medicine. IEEE Trans Med Imaging.

[14] Drokin I, Ericheva E, Lian C, Cao X, Rekik I, Xu X, Yan P (2021). Machine Learning in Medical Imaging: 12th International Workshop, MLMI 2021.

[15] Ebner L, Roos JE, Christensen JD, Dobrocky T, Leidolt L, Brela B (2016). Maximum-Intensity-Projection and Computer-Aided-Detection Algorithms as Stand-Alone Reader Devices in Lung Cancer Screening Using Different Dose Levels and Reconstruction Kernels. AJR Am J Roentgenol.

[16] Drokin I, Ericheva E (2020). Lecture Notes in Computer Science. Social Networks and Texts.

[17] Wang B, Qi G, Tang S, Zhang L, Deng L, Zhang Y (2018). International Conference on Medical Image Computing and Computer-Assisted Intervention.

[18] Li Y, Fan Y (2020). DeepSEED: 3D Squeeze-and-Excitation Encoder-Decoder Convolutional Neural Networks for Pulmonary Nodule Detection. Proc IEEE Int Symp Biomed Imaging.

[19] Perandini S, Faccioli N, Zaccarella A, Re T, Mucelli RP (2010). The diagnostic contribution of CT volumetric rendering techniques in routine practice. Indian J Radiol Imaging.

[20] Cui S, Ming S, Lin Y, Chen F, Shen Q, Li H (2020). Development and clinical application of deep learning model for lung nodules screening on CT images. Sci Rep.

[21] Khosravan N, Bagci U (2018). Medical Image Computing and Computer Assisted Intervention-MICCAI 2018: 21st International Conference, Granada, Spain, September 16-20, 2018, Proceedings, Part II 11.

[22] Cao H, Liu H, Song E, Ma G, Xu X, Jin R (2020). A Two-Stage Convolutional Neural Networks for Lung Nodule Detection. IEEE J Biomed Health Inform.

[23] Chelala L, Hossain R, Kazerooni EA, Christensen JD, Dyer DS, White CS (2021). Lung-RADS Version 1.1: Challenges and a Look Ahead, From the AJR Special Series on Radiology Reporting and Data Systems. AJR Am J Roentgenol.

[24] González Maldonado S, Delorme S, Hüsing A, Motsch E, Kauczor HU, Heussel CP (2020). Evaluation of Prediction Models for Identifying Malignancy in Pulmonary Nodules Detected via Low-Dose Computed Tomography. JAMA Netw Open.

[25] Huang P, Lin CT, Li Y, Tammemagi MC, Brock MV, Atkar-Khattra S (2019). Prediction of lung cancer risk at follow-up screening with low-dose CT: a training and validation study of a deep learning method. Lancet Digit Health.

[26] Rajpurkar P, Irvin J, Ball RL, Zhu K, Yang B, Mehta H (2018). Deep learning for chest radiograph diagnosis: A retrospective comparison of the CheXNeXt algorithm to practicing radiologists. PLoS Med.

[27] Ardila D, Kiraly AP, Bharadwaj S, Choi B, Reicher JJ, Peng L (2019). End-to-end lung cancer screening with three-dimensional deep learning on low-dose chest computed tomography. Nat Med.

[28] Armato SG, Li F, Giger ML, MacMahon H, Sone S, Doi K (2002). Lung cancer: performance of automated lung nodule detection applied to cancers missed in a CT screening program. Radiology.

[29] Wormanns D, Fiebich M, Saidi M, Diederich S, Heindel W (2002). Automatic detection of pulmonary nodules at spiral CT: clinical application of a computer-aided diagnosis system. Eur Radiol.

[30] Brown MS, Goldin JG, Suh RD, McNitt-Gray MF, Sayre JW, Aberle DR (2003). Lung micronodules: automated method for detection at thin-section CT—initial experience. Radiology.

[31] Goo JM, Lee JW, Lee HJ, Kim S, Kim JH, Im JG (2003). Automated lung nodule detection at low-dose CT: preliminary experience. Korean J Radiol.

[32] Yuan R, Vos PM, Cooperberg PL (2006). Computer-aided detection in screening CT for pulmonary nodules. AJR Am J Roentgenol.

